# Does electroacupuncture benefit mixed urinary incontinence? A systematic review and meta-analysis with trial sequential analysis

**DOI:** 10.1007/s00192-021-05057-6

**Published:** 2022-01-28

**Authors:** Yang Cui, Quan Li, Delong Wang, Rui Bao, Limiao Li, Jiamin Zhu, Jianuo Li, Zhuxin Li, Jiantao Yin, Xinyu Zhou, Hongna Yin, Zhongren Sun

**Affiliations:** 1 Heilongjiang University of Chinese Medicine, Harbin, 150040 China; 2 The First Affiliated Hospital of Heilongjiang University of Chinese Medicine, Harbin, 150040 China; 3 The Second Affiliated Hospital of Heilongjiang University of Chinese Medicine, Harbin, 150001 China; 4 The Affiliated Hospital of Nantong University, Nantong, 226001 China

**Keywords:** Electroacupuncture, Mixed urinary incontinence, Systematic review, Meta-analysis, Trial sequential analysis

## Abstract

**Introductin and hypothesis:**

Mixed urinary incontinence (MUI) comprises a combination of urgency and stress. The efficacy and safety of electroacupuncture (EA) for the treatment of MUI remain unclear.

**Objective:**

To assess the efficacy and safety of EA in treating MUI.

**Methods:**

We searched PubMed, CENTRAL, Embase, Web of Science, four Chinese databases, clinical research registration platforms, grey literature, and the reference lists of the selected studies. Risk of bias and quality were evaluated using the Revman 5.4 and Jadad scores. Meta-analysis was performed using Stata 15.1 software. Trial sequential analysis (TSA) was used to assess the stability of the results.

**Results:**

Eight randomized controlled trials comprising 847 patients were included. The meta-analysis results showed that compared with antimuscarinic drugs plus pelvic floor muscle training, EA resulted in significantly less pad weight on the 1-h pad test and statistically significantly lower severity scores on the International Consultation on Incontinence Questionnaire Short Form. The change in the 72-h incontinence episode frequency difference was not statistically significant, and there was no outcome of overall response rate and quality of life in this meta-analysis. Few adverse events occurred in the EA group. The TSA results suggested that the result of change from baseline in the 1-h pad test was stable and the evidence was conclusive.

**Conclusions:**

EA could be a potential treatment option for MUI and is relatively safe. Nevertheless, because of the limitations of this study, our conclusions should be interpreted with caution, and further studies are needed to confirm the comprehensive clinical efficacy and placebo effect of EA.

## Introduction

Mixed urinary incontinence (MUI), a combination of stress and urge incontinence, is characterized by complaints of involuntary loss of urine associated with urgency, exertion, effort, sneezing, or coughing [1, 2]. The prevalence of urinary incontinence in women is estimated at 25% to 45% while that of MUI at 20% to 36% [3, 4]. Moreover, at least half of women with MUI do not report the problem to their doctors [5]. Women with MUI generally have more severe symptoms and respond poorly to treatment compared to those with pure stress urinary incontinence (SUI) or urgency urinary incontinence (UUI) [6]; in addition, the severity of incontinence increases with the duration of MUI [7]. Although MUI is not directly life-threatening, it seriously impacts patient daily behaviour, social interactions, and mental health [8, 9].

Currently, the management of MUI remains a challenge. The initial treatment of MUI is usually conservative; if basic conservative treatment does not help with symptoms, the last resort is surgery [10]. Common conservative treatments include behavioural therapy, pelvic floor muscle training (PFMT), pharmaceutical therapies, and, less commonly, neuromodulation and continence pessary. Behaviour therapy includes dietary changes, such as elimination of caffeine or other bladder irritants, and fluid management. PFMT is recommended as the first-line treatment to reduce urine leakage with stress-predominant symptoms [11]. Pharmaceutical therapy includes antimuscarinic drugs (e.g. solifenacin [12], tolterodine [13], and oxybutynin [14]) and β_3_ agonists, which both reduce urgency-predominant symptoms in MUI [15, 16] and vaginal oestrogen for post-menopausal women with MUI [17]. Surgical treatment is common, with 20% of women in the US undergoing surgery for either stress or mixed incontinence by the age of 80 years [18], and approximately 15% of women will not improve sufficiently after surgery [10]. However, these treatments have the following limitations: (1) the evidence of the efficacy of behaviour modification therapy for MUI is unclear [4]; (2) there are no high-quality data to support the long-term efficacy of PFMT [10]; (3) patients have poor long-term compliance especially with antimuscarinic drugs that could cause adverse events (e.g. cognitive dysfunction [19] and dry mouth [20]); (4) there is lack of evidence of the optimal dose, therapy duration, and long-term efficacy of oestrogen for MUI [10, 11]; (5) when patients with MUI have persistent urgency incontinence following SUI type surgery, they consider that the procedure has failed [21]. Is there a treatment that satisfies both components of MUI and is it safer and less invasive?

Electroacupuncture (EA) is an important part of traditional Chinese medicine; here, the poles of the wire are connected to the handle or body of the acupuncture needle handle or body, and subsequently output pulse current on the acupoint through the EA instrument. The EA stimulation intensity can be accurate to 0.1 mA, and there are three consecutive wave forms: consecutive, spare-dense, and intermittent waves. The frequency of consecutive waves can be adjusted between 1 to 100 Hz; frequencies < 30 Hz are called sparse waves, and those ≥ 30 Hz are called dense waves. A spare-dense wave is a combination wave with frequencies automatically alternating between those of sparse and dense waves; it has an alternating time of approximately 1.5 s at which frequency is fixed at 4 Hz and 20 Hz. An intermittent wave is a combination wave that automatically appears rhythmically and intermittently, with no current and continuous dense wave interconversion with an alternating time of approximately 1.5 s. EA has been widely used for the prevention and treatment of various diseases and has a quick onset of action, low rate of relapse, and minor adverse reactions [22–24]. According to a previous study [25], EA may regulate and enhance sacral and pelvic floor muscle function, suggesting that EA may play a positive role in treating MUI symptoms [26]. In recent years, clinical reports on EA for MUI have shown an increasing trend, and four clinical guidelines have discussed acupuncture (including EA) in the management of urinary incontinence; however, there is insufficient evidence of the efficacy of EA [27]. Moreover, no study has objectively evaluated and analysed the effectiveness and safety of EA in the treatment of MUI from the perspective of evidence-based medicine.

Consequently, to address the above inconsistencies and provide an evidence-based medical basis for EA therapy for MUI, this study adopted a systematic review and meta-analysis design with trial sequential analysis (TSA) to objectively and comprehensively evaluate randomized controlled trials (RCTs) of EA for MUI. The conclusions obtained by sequential analysis are more reliable than those obtained by traditional meta-analysis in that TSA can reduce false-positive results caused by random errors and repeated significance tests in meta-analysis [28–30]; therefore, we applied TSA to the results of the meta-analysis.

## Methods

This present study was strictly implemented in accordance with the Preferred Reporting Items for Systematic Reviews and Meta-Analyses (PRISMA) reporting guidelines [31] and was registered on PROSPERO; the ID number is CRD42020220528.

### Search strategy

PubMed, Embase, the Cochrane Central Register of Controlled Trials (CENTRAL), Web of Science, China National Knowledge Infrastructure, China Biomedical, Chongqing VIP Database, and Wanfang Database were searched. The two researchers searched articles independently using the following search terms: “Urinary Incontinence”; “mixed urinary incontinence”; “mixed incontinence”; “Acupuncture”; “Acupuncture Therapy”; Electroacupuncture”; “acupuncture”; “electroacupuncture therapy”. The retrieval time was from the date of inception of the database to 1 June 2021. The complete search strategy for the Cochrane Library is presented in Table 1. The search strategy was applied to other databases. Additionally, the clinical research registration platform (WHO International Clinical Trial Registration platform and China Clinical Trial Registration Center) and grey literature were also searched. Meanwhile, the two researchers searched the studies independently, cross-checked their results with each other, and then searched the reference lists of the selected studies.

**Table 1:** CENTRAL: sessionresults

Search strategy (CENTRAL database)		
Number	Search terms	Results
*#*1	MeSH descriptor: [Urinary Incontinence] explode all trees	2311
*#*2	(mixed urinary incontinence): ti, ab, kw	616
*#*3	(mixed incontinence): ti, ab, kw	685
*#*4	*#*1 OR *#*2 OR *#*3	2777
*#*5	MeSH descriptor: [Acupuncture] explode all trees	151
*#*6	MeSH descriptor: [Acupuncture Therapy] explode all trees	4855
*#*7	MeSH descriptor: [Electroacupuncture] explode all trees	825
*#*8	(acupuncture): ti, ab, kw	15,261
*#*9	(electroacupuncture): ti, ab, kw	2622
*#*10	(electroacupuncture therapy): ti, ab, kw	1490
*#*11	*#*5 OR *#*6 OR *#*7 OR *#*8 OR *#*9 OR *#*10 OR *#*11	16,160
*#*12	*#*4 AND *#*11 (in trials)	46

*ti*   title; *ab*   abstract; *kw*   keywords

### Inclusion and exclusion criteria

#### Inclusion criteria

A study was included if it met all five of the following criteria:

1. Type of study design

Only RCTs evaluating the efficacy of EA for MUI were included. The language was restricted to English and Chinese.

(2) Types of participants

Women (age ≥ 18 years) who were clearly diagnosed with MUI according to the diagnostic criteria outlined in the European Association of Urology Working Panel: EAU guidelines on urinary incontinence [32] or Chinese Society of Urological Surgery: Guidelines for the Diagnosis and Treatment of Urinary Incontinence [33], according to the patient’s symptoms of involuntary loss of urine associated with urgency and exertion, effort, sneezing, coughing, or physical examination, etc.

(3) Types of the treatment group

The treatment included either EA alone or EA in combination with other therapies. When EA was administered in combination with other therapies, the therapy administered to the EA group had to also be administered to the control group, and other types of acupuncture were not included.

(4) Types of control group

The control group consisted of patients who received conservative therapy (e.g. pelvic floor muscle training, bladder training), effective pharmacotherapy (e.g. solifenacin and tolterodine), or sham electroacupuncture, which was used alone or in combination.

(5) Types of outcome indicators

A study was included if contained at least one of the following outcome indicators:

- Primary outcome, which was the change from baseline in the 1-h pad test [26, 34].
- Secondary outcomes, which were the:
  (i) Change from baseline of International Consultation on Incontinence Questionnaire Short Form (ICIQ-SF) scores;
  (ii) Change from baseline in the 72-h incontinence episode frequency (IEF);
  (iii) Response rate;
  (iv) Quality of life;
  (v) Adverse events.

#### Exclusion criteria

A study was excluded if:

1. it was a secondary analysis or duplicate publication (with multilingual publications, only the earliest was chosen).
2. the full text could not be obtained.
3. the design was flawed, such as studies without clear random grouping methods or not strictly following the randomisation principle (e.g. randomisation performed based on the order in which patients were admitted to the hospital or odd-even numbering of their medical record number or date of birth).
4. the baseline data were incomplete or if the study had no baseline data evaluation.
5. acupuncture techniques other than EA alone were used in the study.

### Trials selection

After removing the duplicate publications using the literature management software (EndNote X9), all headings and abstracts were independently reviewed by the two researchers according to the inclusion and exclusion criteria to retrieve and determine eligible studies. The studies that did not meet the criteria were initially excluded; subsequently, the full text of the studies whose titles and abstracts met the inclusion criteria were downloaded and carefully read to determine whether they could be included. The two researchers resolved any difference of opinion through discussion. If no agreement could be reached, they consulted with an arbiter for a decision. All researchers were registered traditional Chinese medicine practitioners with at least 2 years of clinical acupuncture experience.

### Data extraction

After determining the studies to be included, we prepared a pre-set data template. The two researchers separately extracted the data from the template. The data included study characteristics (first author, year of publication), participant characteristics (age, sample size, and duration of MUI), interventions (type, treatment duration, frequency, and acupoint), diagnostic criteria, outcome measures, and adverse events. When differences arose, they were resolved through discussion, and if opinions differed, an arbitrator was consulted for a decision.

### Risk of bias and quality assessment

According to the Cochrane Collaboration recommendation [35], all included studies should be evaluated for the following seven major risks of bias: random sequence generation (selection bias), allocation hiding (selection bias), blinding of participating researchers (performance bias), blinding of result evaluation (detection bias), incomplete result data (attrition bias), selective reporting (reporting bias), and other biases. The two researchers independently evaluated all included studies using the Cochrane Collaboration tool (Revman version 5.4) at three grades of bias (high, low, and unclear). Disputes were resolved through discussion; when opinions differed, they consulted an arbiter for a decision. The analysed outcomes were determined and illustrated with the support of Review Manager.

The two researchers independently evaluated the quality of the included studies using the Jadad score; in case of a difference of opinion, they consulted an arbiter for a decision. The main evaluation was of the following three aspects (1–5 points): (1) generation method of the random grouping sequence: a random sequence generated by computer or by a random number table (2 points); random assignment mentioned in the experiment, but the method of generating the random sequence was not explained (1 point); semi-randomized trials or random method error trials, such as order of admission and odd or even number of birth dates (0 points). (2) Double-blind method: the specific method of double-blind implementation was described and considered appropriate (2 points). Only the double-blind method was mentioned (1 point). The trial mentioned the use of the double-blind method, but the method was not appropriate (0 points). (3) Withdrawal and loss to follow-up: the number of cases of withdrawal and loss to follow-up and reasons for withdrawal were described in detail (1 point). There was no mention of exit or lost visits (0 points). A score of 1 to 2 points signified a low-quality study and 3 to 5 points signified a high-quality study [36].

### Statistical analysis

Meta-analysis was performed using Stata 15.1 software. Continuous variables were assessed using weighted mean differences (WMDs) and corresponding 95% confidence intervals (CIs) for the effectiveness analysis of statistics; a difference of < 0.05 was considered statistically significant. Dichotomous variables are expressed as relative risk (RR) and corresponding 95% CIs. RR > 1 indicated high efficacy in the treatment group; RR < 1 indicated high efficacy in the control group; RR = 1 indicated that the difference was not statistically significant between the two groups.

Heterogeneity was assessed using the I^2^ test. If I^2^ was ≤ 50%, the heterogeneity was not significant, and the fixed-effects model was used for the meta-analysis; I^2^ > 50% indicated significant heterogeneity, and a random-effects model was used for the meta-analysis. If an outcome indicator contained more than nine studies, meta-regression analysis was used to clarify the source of heterogeneity among studies; in contrast, subgroup and sensitivity analyses were performed. The objective of this study was to evaluate the effectiveness of EA as a treatment for MUI. Therefore, the subgroup analysis was mainly determined based on the type of intervention. Sensitivity analysis was performed by eliminating each data point to evaluate the stability of the results. If an outcome indicator contained more than nine studies, funnel plots were drawn to evaluate publication bias; in contrast, Egger’s test was used to quantify the significance of publication bias if an outcome indicator contained fewer than nine studies (with *p* < 0.05 considered significant). If the outcome showed the data of mean and standard error (SE), the standard deviation (SD) was calculated as $\mathrm{SD}=\mathrm{SE}\times \sqrt{n}\left(\mathrm{n}=\mathrm{sample}\ \mathrm{size}\right)$ [35].

### Trial sequential analysis

TSA was used to estimate the sample size of the meta-analysis and overcome the shortcomings of classical meta-analysis. Meta-analysis usually requires the accumulation of multiple trial results, thus increasing random errors. Some “positive” meta-analysis results may be attributable to random error; when the number of trials in a meta-analysis and the sample size of patients are small, random error may yield erroneous results [28–30]. Therefore, it is important to apply TSA to minimize the risk of false-positive or false-negative results in a meta-analysis. In this study, the researchers used the version 0.9.5.10 Beta TSA software to perform the analysis, calculating the required information size (RIS), referring to the number of cases needed to achieve statistically significant differences in the meta-analysis. Concurrently, the alpha spending function, continuously monitoring boundaries, and evaluation of invalid boundary areas were estimated [37].

## Results

### Literature retrieval

**Fig. 1:**
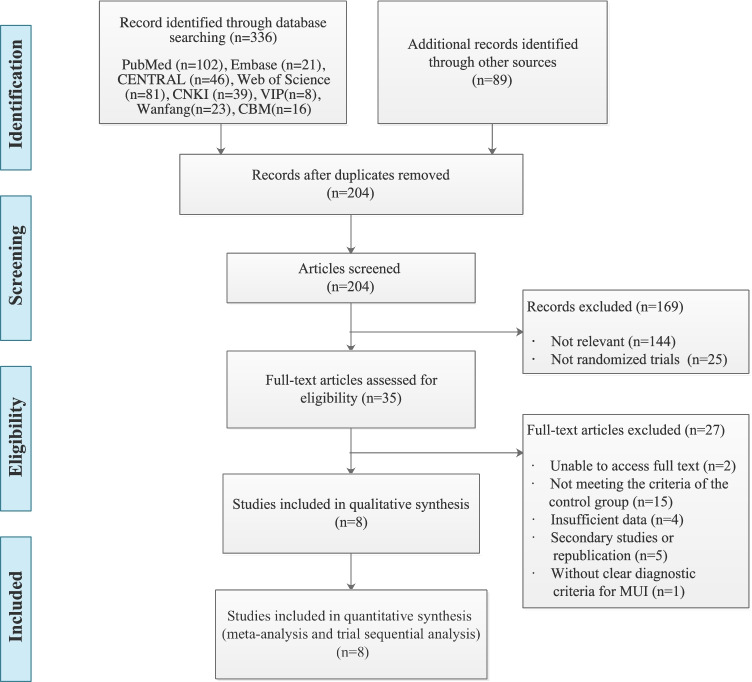
Flow chart of study selection

A preliminary search identified 425 records from eight databases and other sources including grey literature and the reference lists of the selected studies. A total of 204 studies were selected after duplicates were removed, with 144 unrelated studies and 25 non-RCTs. After reading the titles and abstracts, 35 studies were selected. After reading the full text of the selected articles, 2 studies whose full texts could not be obtained, 15 studies that did not meet the inclusion criteria of the control group, 4 studies that had missing baseline data, 5 studies that were republished, and 1 study that did not have clear diagnostic criteria were excluded. Ultimately, a total of eight RCTs [38–45] that met the criteria were included in the statistical analysis (Fig. 1).

### Studies characteristics

All studies were conducted in China, with one published in English [38] and the other seven in Chinese [39–45]. All studies were published in or after 2015, with a total of 847 patients and 16 intervention groups, and ages ranging from 29 to 75 years; the baseline data for each study were comparable. All included studies were peer-reviewed and included five dissertations [39, 40, 42, 44, 45]. Interestingly, all treatment and control groups in the included studies received single EA therapy versus antimuscarinic drugs combined with PFMT. For details, see Table 2.

**Table 2:** Characteristics of the included studies

Author and year		EA group				Control group						
	Age	Sample size	MUI (y) duration	Type	Acupoints^**&**^	Sample size	MUI (y) duration	Type	Treatment period*	Diagnostic criteria	Outcome measures	Adverse events (n)
Liu et al. 2019 [38]	35–75	249	3.1 ± 4.7	EA (spare-dense wave, 10/50 Hz, 0.1 to 5.0 mA, 30 min)	BL33, BL35	248	4.0 ± 3.6	Solifenacin plus PFMT	12 wks (3 per wk)	(1)	△①②③	EA group, subcutaneous hematoma (10), digestive system (4), others (27); solifenacin-PFMT, severe events (1), digestive system (70), others (20)
Chen 2016 [39]	38–75	25	9.0 ± 8.1	EA (spare-dense wave, 50 Hz, 0.1 to 5.0 mA, 30 min)	BL33, BL35	25	10.5 ± 7.9	Solifenacin plus PFMT	12 wks (3 per wk)	(2) (3)	△①②	Solifenacin-PFMT group, slightly dry mouth (7)
Shi 2015 [40]	46–75	30	8.7 ± 2.7	EA (spare-dense wave, 10/50 Hz, 0.1 to 5.0 mA, 30 min)	BL33, BL35, BL23, SP6	30	9.0 ± 1.7	Solifenacin plus PFMT	12 wks (3 per wk)	(2) (3)	①③	NR
Wang et al. 2017 [41]	36–74	23	NR	EA (spare-dense wave,10/50 Hz, NR, 30 min)	BL33, BL35	22	NR	Solifenacin plus PFMT	12 wks (3 per wk)	(2)	△①②	NR
Wang 2016 [42]	35–72	25	NR	EA (spare-dense wave, 5 Hz, 0.1 to 5.0 mA, 30 min)	BL33, BL35	25	NR	Solifenacin plus PFMT	12 wks (3 per wk)	(2) (3)	△①②	Solifenacin-PFMT group, significant dry mouth (2)
Hong et al. 2015 [43]	29–69	32	8.0 ± 5.8	EA (consecutive wave, 20/30 Hz, NR, 30 min)	RN3, RN4, ST36, SP6	33	7.6 ± 5.5	Tolterodine plus PFMT	4 wks (5 per wk)	(2) (4)	△①③	EA group, subcutaneous hematoma (1)
Zhang SW 2015 [44]	35–75	21	6.1 ± 5.2	EA (spare-dense wave, 10/50 Hz, 0.1 to 5.0 mA, 30 min)	BL33, BL35	20	8.1 ± 7.5	Solifenacin plus PFMT	12 wks (3 per wk)	(2) (3)	△①②③	Solifenacin-PFMT group, dry mouth (5), dry eye (2)
Zhang SN 2015 [45]	35–75	20	7.2 ± 6.1	EA (spare-dense wave, 10/50 Hz, NR, 30 min)	BL33, BL35	19	7.5 ± 7.0	Solifenacin plus PFMT	3 per wk (12 wks)	(2) (3)	②③	Solifenacin-PFMT group, slightly dry mouth (5)

*RCTs*   randomized controlled trials; *MUI*   mixed urinary incontinence; *EA*   electroacupuncture; *PFMT*   pelvic floor muscle training; *NR*   non-related; *&*   bilateral acupuncture points; *   EA treatment times a week; (1) European Association of Urology Working Panel: EAU guidelines on urinary incontinence (2011); (2) The International Consultation on Urological Diseases (ICUD): INCONTINENCE (4th Edition 2009); (3) Chinese Society of Urological Surgery: Guidelines for the Diagnosis and Treatment of Urinary Incontinence (2007); (4) Guidelines for Diagnosis and Treatment of Urological Diseases in China (2014); △ = change from baseline of 1-h pad test; ① = change from baseline of ICIQ-SF scores; ② = change from baseline of 72-h IEF; ③ = total clinical effective rate

### Study quality assessment and risk of bias

**Fig. 2:**
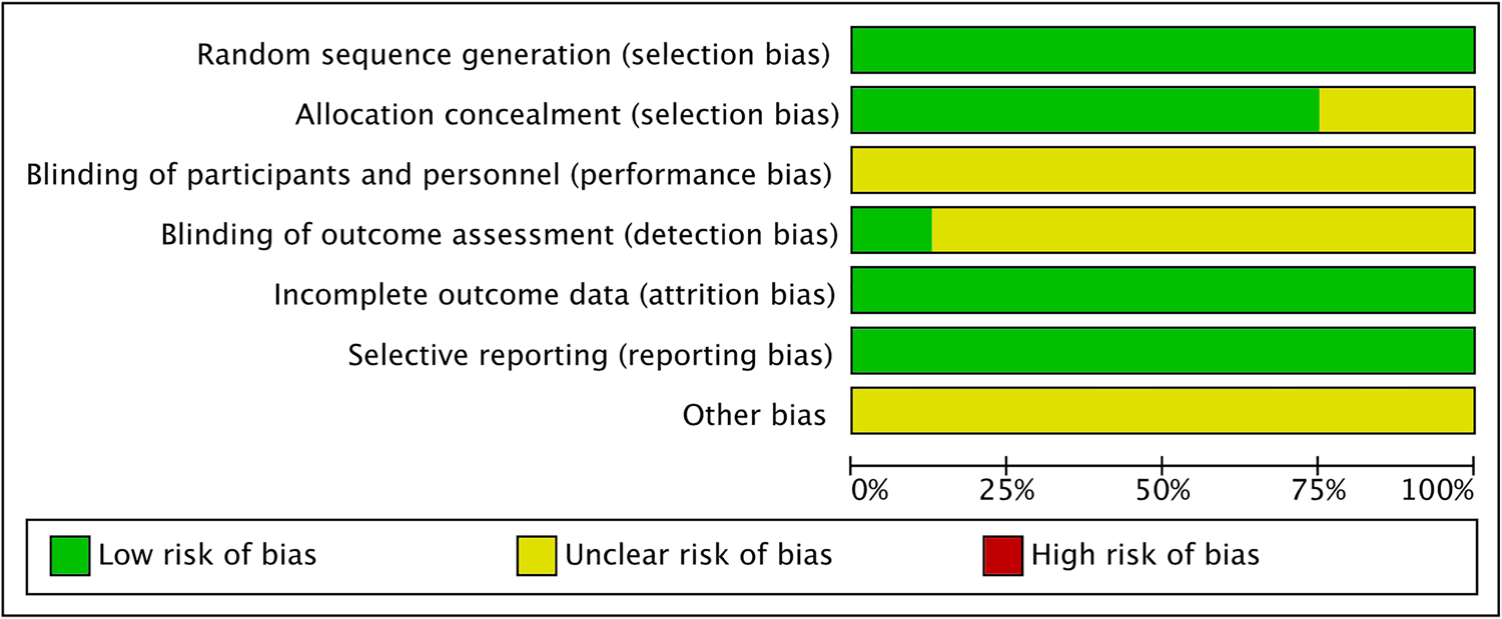
Percentage graph showing the risk of bias

**Fig. 3:**
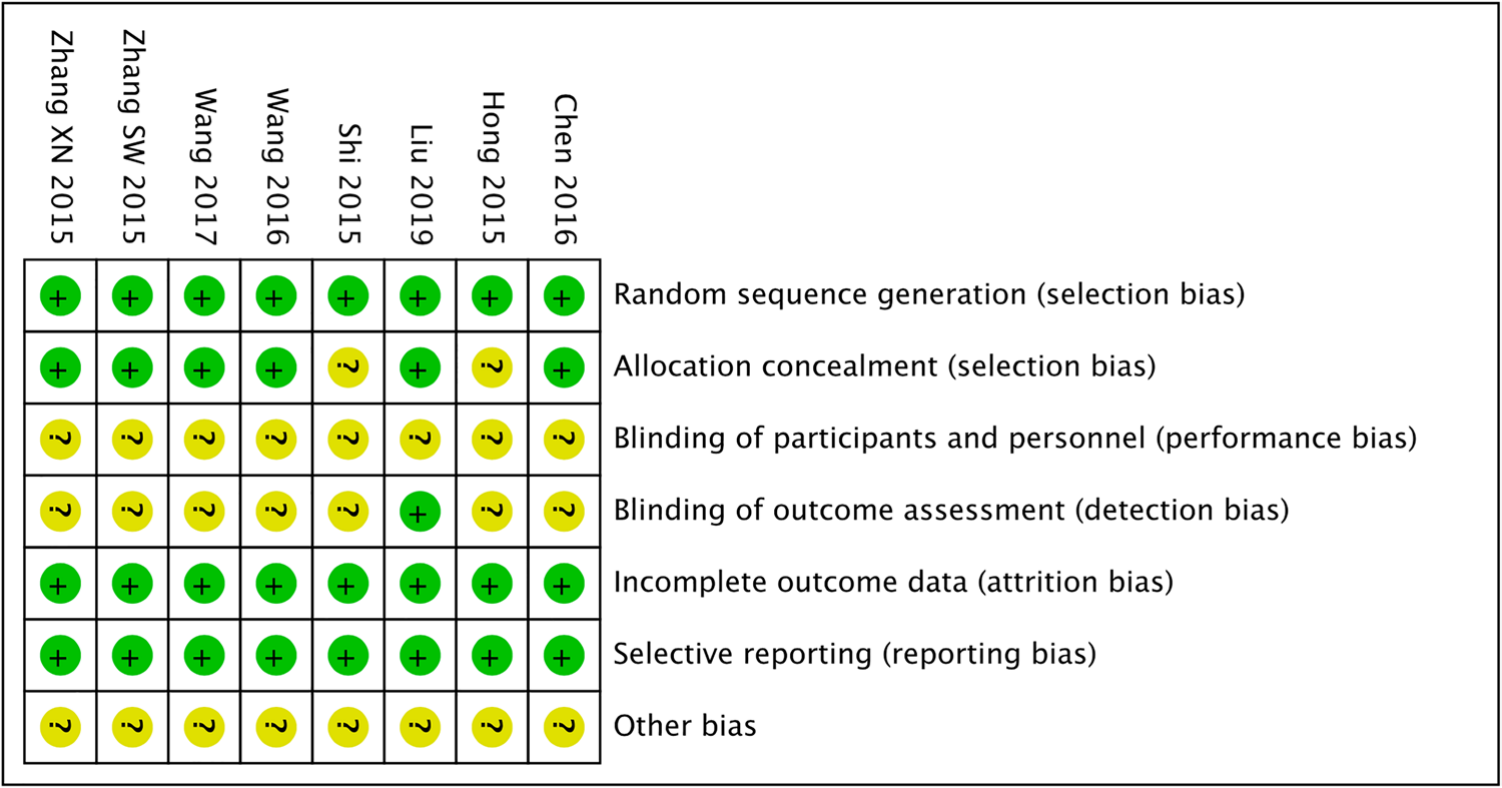
Summary graph showing the risk of bias

All eight studies mentioned random sequence generation; two studies used the random number table method [40, 43], and six used a central randomisation system [38, 39, 41, 42, 44, 45]. Six studies [38, 39, 41, 42, 44, 45] used the central randomisation system method for allocation concealment, while two did not describe the allocation concealment method. None of the studies described how the subjects were blinded. In terms of result evaluator blindness, one study [38] described the concrete method, while the remaining seven studies did not mention whether the evaluator was blinded or designed. Six studies reported data and the specific reasons for the loss of follow up [38, 39, 42–45]. All eight studies reported the outcome indicator results. Other biases were not clear in any of the studies. Regarding the quality assessment of the studies included, EA therapy, because of its peculiarity, was difficult to camouflage and hide from blinded physicians and patients; however, the overall quality of the research included was moderate. Six and two studies were of moderate and low quality, respectively. Table 3 and Figs. 2 and 3 summarize the quality assessment and risk of bias of all included studies.

**Table 3:** Risk of bias and quality assessment of the included studies

Author and year	Sequence generation	Allocation concealment	Blinded method	Outcome blinded	Result data integrity	Selective reporting	Other bias	Jadad score
Liu et al. 2019 [38]	Central randomisation system, low	Low	Unclear	Low	Low	No, low	Unclear	3
Chen et al. 2016 [39]	Central randomisation system, low	Low	Unclear	Unclear	Low	No, low	Unclear	3
Shi et al. 2015 [40]	Random number table, low	Unclear	Unclear	Unclear	Low	No, low	Unclear	2
Wang et al. 2017 [41]	Central randomisation system, low	Low	Unclear	Unclear	Low	No, low	Unclear	2
Wang et al. 2016 [42]	Central randomisation system, low	Low	Unclear	Unclear	Low	No, low	Unclear	3
Hong et al. 2015 [43]	Random number table, low	Unclear	Unclear	Unclear	Low	No, low	Unclear	3
Zhang et al. 2015 [44]	Central randomisation system, low	Low	Unclear	Unclear	Low	No, low	Unclear	3
Zhang et al. 2015 [45]	Central randomisation system, low	Low	Unclear	Unclear	Low	No, low	Unclear	3

### Meta-analysis findings

#### Change from baseline in the 1-h pad test

**Fig. 4:**
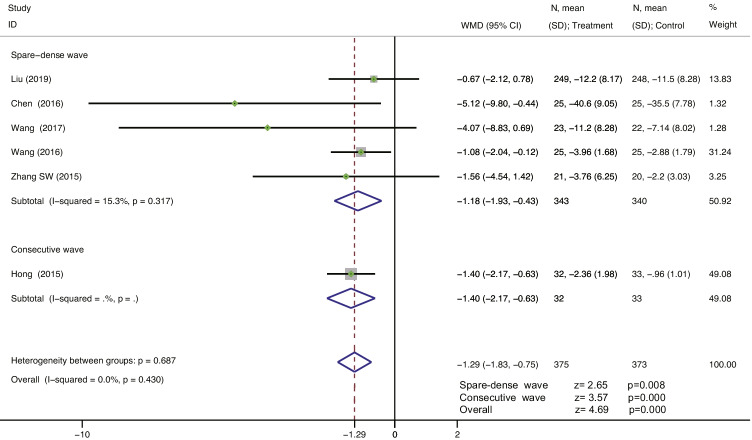
Forest plot for treatment and control group. Change from baseline in the 1-h pad test

**Fig. 5:**
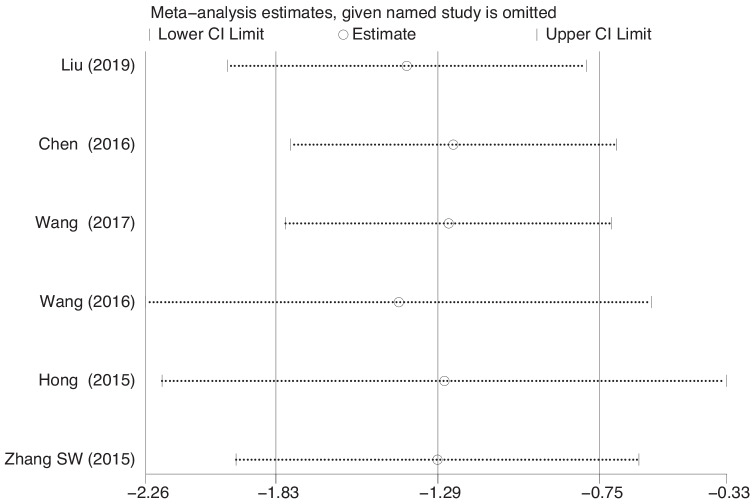
Sensitivity analysis figure. Change from baseline in the 1-h pad test

Six studies [38, 39, 41–44] reported the change in the amount of urine leakage from baseline in the 1-h pad test in 748 patients. The heterogeneity between studies was I^2^ = 0.0%, indicating that the studies had good homogeneity. A fixed-effects model was selected for the analysis. The results showed that EA therapy was superior to the combination of antimuscarinic drugs with PFMT in improving the amount of urine leakage in the 1-h pad test in MUI; the difference was statistically significant (WMD = −1.29, 95% CI = −1.83 to −0.75, *P* = 0.000 < 0.001). For the spare-dense wave of EA therapy vs. antimuscarinic drugs plus PFMT, the subgroup analysis in five studies showed statistically significant differences (WMD = −1.18, 95% CI = −1.93 to −0.43, *P* = 0.008 < 0.05). For the consecutive wave of EA treatment vs. antimuscarinic drugs plus PFMT, the subgroup analyses in one study showed statistically significant differences (WMD = −1.40, 95% CI = −2.17 to −0.63, *P* = 0.000 < 0.001) (Fig. 4). The sensitivity analysis showed that there was little or no change in the results, by omitting one study at a time (Fig. 5).

#### Change from baseline in the ICIQ-SF scores

**Fig. 6:**
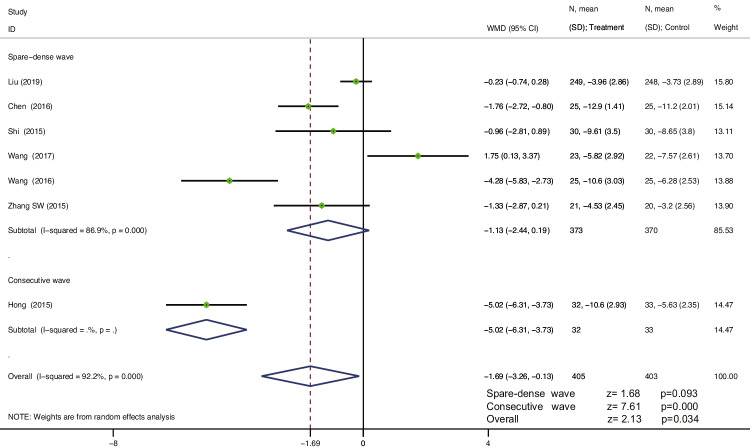
Forest plot for the treatment and control group. Change from baseline in the ICIQ-SF scores

Seven studies [38–44] reported a change from baseline in the ICIQ-SF scores, including 808 patients. The heterogeneity between studies was I^2^ = 92.2%, and a random-effects model was used to analyse the effect size of each study’s data. The results showed that EA treatment was superior to antimuscarinic drugs combined with PFMT for MUI in reducing the ICIQ-SF scores, and the difference was statistically significant (WMD = −1.69, 95% CI = −3.26 to −0.13, *P* = 0.034 < 0.05). For the spare-dense wave of EA treatment vs. antimuscarinic drugs plus PFMT, the subgroup analysis in six studies showed no statistically significant differences (WMD = −1.13, 95% CI = −2.44 to 0.19, *P* = 0.093 > 0.05). For the consecutive wave of EA treatment vs. antimuscarinic drugs plus PFMT, the subgroup analysis in one study showed statistically significant differences (WMD = −5.02, 95% CI = −6.31 to −3.73, *P* = 0.000 < 0.001) (Fig. 6). The sensitivity analysis showed that there was little or no change in the results by omitting one study at a time (see Appendix).

#### Change from baseline in the 72-h IEF

**Fig. 7:**
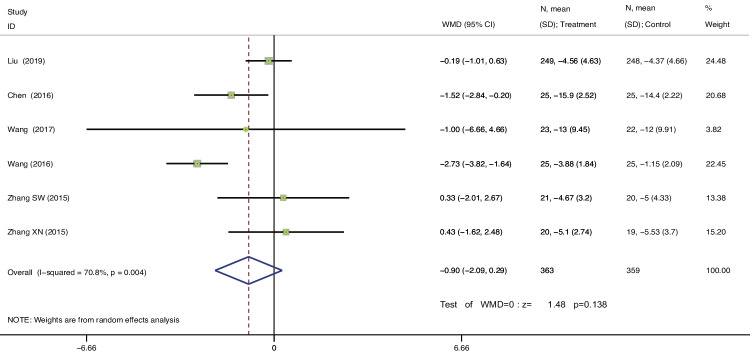
Forest plot for the treatment and control groups. Change from baseline in the 72-h IEF

As shown in Fig. 7, six studies [38, 39, 41, 42, 44, 45] reported a change from baseline in the 72-h IEF. The heterogeneity between studies was I^2^ = 70.8%; therefore, the random-effects model was selected. The results showed that EA therapy vs. antimuscarinic plus PFMT for MUI played a role in reducing 72-h IEF; the difference was not statistically significant (WMD = −0.90, 95% CI = −2.09 to 0.29, *P* = 0.138 > 0.05). As there were spare-dense waves only in the EA group, subgroup analysis was not performed. The sensitivity analysis showed that there was little or no change in the results by omitting one study at a time (see Appendix).

#### EA treatment for MUI on the response rate

Five studies [38, 40, 43–45] reported a response rate for 683 patients. However, the efficacy criteria for these studies were entirely self-designed, and the criteria for clinical efficacy or improvement were inconsistent in each study. For instance, Liu et al., observed participant global impression improvement measured on a 7-point Likert scale (marked worsening to marked improvement) [38], and Shi judged whether EA was effective by observing whether the 1-h pad test was positive and monitoring the patient’s self-feel urine leakage [40]. Hong et al. determined the efficacy by measuring the degree of change in the ICIQ-SF scores and urine leakage with the 1-h pad test [43]. Therefore, no data summaries or meta-analyses were performed.

### Publication Bias

Because the change from baseline in the 1-h pad test of the outcome measure was fewer than nine items in the included studies, the funnel plot publication bias test could not be performed. Egger’s test was used to quantify the significance of publication bias. The result showed that *P* = 0.16 > 0.05, indicating that there was no significant risk of publication bias (Table 4).

**Table 4:** Publication bias

Number of studies = 6					Root MSE = 0.8367	
Std_Eff|	Coef.	Std. err.	t	P > |t|	95% conf. interval	
slope |	−0.6921441	0.414614	−1.67	0.170	−1.843297	0.459009
bias |	−1.063698	0.6168045	−1.72	0.160	−2.776222	0.648826

*MSE*   mean square error

### Adverse events

Six of the eight studies reported adverse events [38, 39, 42–45]. The remaining two studies did not describe the occurrence of adverse events [41, 42]. All six studies reported adverse events in the control group, and only two studies reported minor adverse events in the EA group [38, 43]. Adverse events in the EA group included subcutaneous haematoma, digestive system symptoms (abdominal pain and diarrhoea), and others. The adverse events in the control group included severe events (one participant had a fracture from an accident), digestive system symptoms (dry mouth, constipation, abdominal pain, diarrhoea, dyspepsia, and heartburn), dry eye, and others such as influenza. Liu et al. [38] reported subcutaneous haematoma (*n* = 10), digestive system symptoms (*n* = 4), and others (*n* = 27) in the EA group and severe events (one participant had a fracture from an accident, *n* = 1), digestive system symptoms (*n* = 70), and others (*n* = 20) in the solifenacin-PFMT group. Chen [39] reported a slightly dry mouth (*n* = 7) in the solifenacin-PFMT group. Wang [42] reported a significantly dry mouth (*n* = 2) in the solifenacin-PFMT group. Zhang SW [44] reported dry mouth (*n* = 5) and dry eye (*n* = 2) in the solifenacin-PFMT group. Zhang XN [45] reported a slightly dry mouth (*n* = 5) in the solifenacin-PFMT group.

### Trial sequential analysis findings

**Fig. 8:**
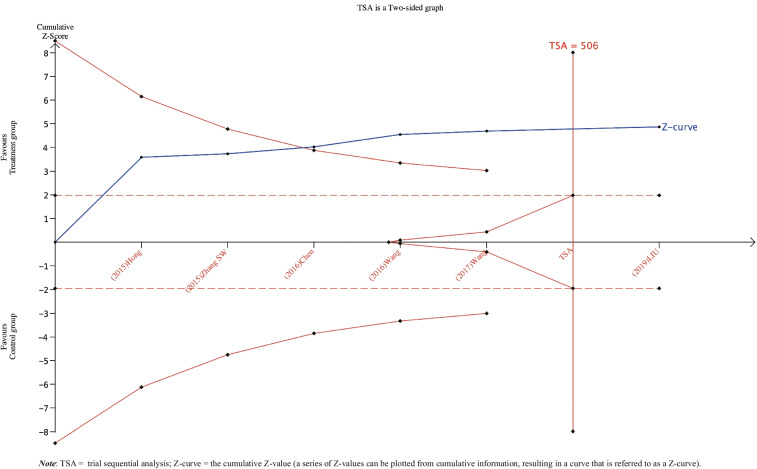
Trial sequential analysis of the 1-h pad test for EA in treating MUI

The TSA results are shown in Fig. 8. Six RCTs including 748 patients were entered into the meta-analysis. The actual RIS sample size required for the meta-analysis was 506. The RIS estimate was based on the following statistical indicators: type I error probability (*α* = 0.05), type II error probability (*β* = 0.2), and relative risk reduction (RRR = 20%). The TSA results showed that the Z-curve crossed the traditional threshold (red dotted line) and the TSA threshold (the solid red line at the top), indicating that a firm and positive conclusion had been obtained before expectations were met; EA therapy was better than the combination of antimuscarinic drugs and PFMT for the reduction of urine leakage in patients with MUI as ascertained with the 1-h pad test. With the concreteness of this evidence, no further study was needed.

## Discussion

Presently, the pathological mechanism and pathogenesis of MUI have not been clarified. The prevailing view is that the pathophysiology of MUI may involve urethral sphincter deficiency, overactivity of the urethra, detrusor overactivity, or a combination of all three [38]. Increasing age is a well-recognized risk factor for MUI in many population-based studies, and other risk factors, including delivery, obesity, diabetes, hypertension, hormonal changes, sedentary behaviour, cigarette smoking, chronic medical conditions, and pelvic conditions, could contribute to MUI [46–49]. This may be related to the underlying pathogenesis of MUI. EA therapy could be a treatment option for MUI; a high-quality clinical study preliminarily confirmed the efficacy of EA in the treatment of SUI [26], suggesting that EA therapy may be an effective complementary and alternative approach in the treatment of MUI.

**Fig. 9:**
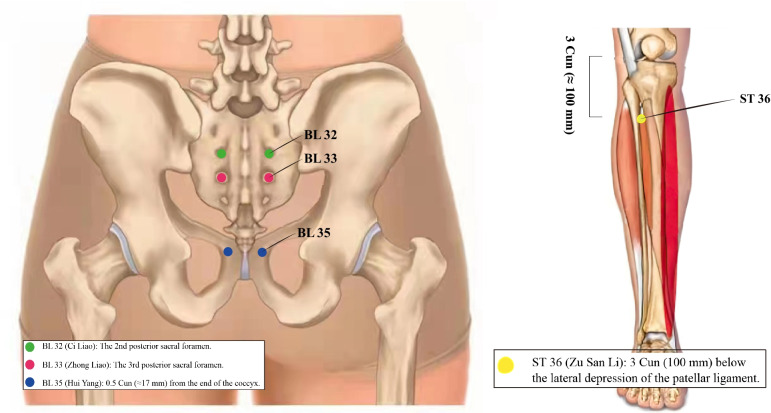
The specific locations of acupoints BL32, BL33, BL35, and ST36

Few studies have examined the mechanism of EA in the treatment of MUI, and animal experiments have suggested that acupuncture of ST36 (Zu San Li) located on the lateral side of the calf, 3 Cun (≈100 mm) below the lateral depression of the patellar ligament, can decrease the muscarinic binding ability of the cerebral cortex, hippocampus, striatum, and spinal cord in rats [50]. EA stimulation of BL33 (Zhong Liao), located at the third posterior sacral foramen, and BL35 (Hui Yang), located 0.5 Cun (≈17 mm) from the end of the coccyx, can increase the concentration of types I and III collagen in the supporting tissue of the pelvic floor of SUI model rats [51, 52]. Other studies have suggested that electroacupuncture BL32 (Ci Liao), located at the second posterior sacral foramen, could downregulate the expression of VR1 in the sacral urination centre of rats and improve the urgent symptoms of incontinence [53, 54]. In summary, there are two main conclusions: on the one hand, it is possible to stimulate the third sacral (S3) nerve through the BL33 point and the pudendal nerve through the BL35 point. S3 and the pudendal nerve, involved in the regulation and contraction of the pelvic floor muscles, similar to PFMT, can improve the function of the pelvic floor muscles, which benefits the stress component of MUI. On the other hand, similar to sacral neuromodulation, EA may also restrain detrusor overactivity to improve the urgency component of MUI. The specific locations of the acupoints are shown in Fig. 9.

To our knowledge, this was the first systematic review and meta-analysis (using TSA) of EA therapy for MUI. In total, 847 patients were included in eight RCTs of EA therapy for MUI. All included RCTs compared EA therapy with antimuscarinic drugs combined with PFMT, and seven studies used the BL33 (Zhong Liao) and BL35 (Hui Yang) acupoints. It is usually recommended that PFMT in combination with antimuscarinic drugs (e.g. solifenacin and tolterodine) should be used for women with MUI [15]. Current research has shown that antimuscarinic drugs are useful for the treatment of UUI and MUI [12, 55], and previous studies have supported that PFMT is the first-line treatment for SUI and MUI [56–58]. Therefore, it is important to compare EA therapy with antimuscarinic drugs plus PFMT. As for the setting of outcome indicators, this study did not identify clinically significant differences among the outcome indicators, but the 1-h pad test is the only test following a standardized procedure for urinary incontinence [59]. The specificity of the 1-h pad test is 65% to 89% [60], and compliance is high [61]; the results of the 1-h pad test also showed a positive correlation with urinary leakage symptoms and quality of life [26]. Therefore, the 1-h pad test was selected as the primary outcome measure in this study.

The results of the meta-analysis based on the existing available research data indicated that compared with the antimuscarinic drugs plus PFMT groups, EA therapy for MUI was superior in reducing urine leakage based on the 1-h pad test and ICIQ-SF scores, and the difference in the reduction in the 72-h IEF was not statistically significant. Regarding the safety analysis, only two of the eight studies mentioned the adverse events of EA therapy, mainly presenting as mild reactions such as subcutaneous haematoma and digestive system reaction, while five studies reported adverse reactions in the antimuscarinic drugs plus PFMT group, presenting as dry mouth, dry eyes, and severe digestive system reactions. Compared with the antimuscarinic drugs plus PFMT group, there were fewer adverse reactions and higher safety in the EA therapy group. The findings of the TSA indicate that the results of the 1-h pad test in this meta-analysis were robust, and these evidence-based results may have implications for clinical practice. TSA is a useful tool for systematic reviews and meta-analyses. Even if the TSA results do not support the deterministic conclusion of the meta-analysis, they can provide a quantitative and visual basis for subsequent studies to indicate the sample size needed to accept or reject an intervention.

Nevertheless, there are some limitations to this study. First, one secondary outcome, that is, changes from baseline in the 72-h IEF, did not support the results of the primary outcome indicators of this study; this may be related to the compliance and accuracy of measurement of the 72-h urination diary. With the high heterogeneity of this result and low quality of evidence, further verification is needed. Second, because of the specificity of EA therapy, different acupuncturists do not follow a unified, standardized treatment protocol; based on their respective techniques and experiences, each physician’s acupuncture technique and penetration depth differ. Third, only eight studies that meet the criteria were included, and the sample size in most studies was small; therefore, the design was not rigorous, and the lack of allocation concealment and use of blind design in many studies resulted in lower methodological quality of the RCTs, such that the power of this finding is limited. In the future, more high-quality studies with reasonable allocation hiding and patient and measurer blinding are needed to improve the quality of evidence. Fourth, this study was unable to determine the response rate of EA therapy because the efficacy criteria of each study were inconsistent. Fifth, only English and Chinese were selected as literature retrieval languages, which increased the risk of selection reporting bias. Finally, according to the available data, the effective boundary of antimuscarinic drugs combined with PFMT for MUI cannot be defined, and the results obtained from this meta-analysis can only indicate that EA therapy is more effective than antimuscarinic drugs combined with PFMT in controlling patient urine leakage and reducing the ICIQ-SF scores, but the placebo effect cannot be excluded.

Future prospects: (1) Animal experimental studies constitute the main method and approach to explain the role of EA in the treatment of MUI, and it is important to explain the mechanism of EA in the treatment of MUI from the perspective of pathophysiology. There have been few such studies, and basic studies on EA therapy for MUI should be carried out in the future to explore the potential use of EA for the treatment of incontinence. (2) Existing studies lack a unified clinical validity standard and assessment of quality of life in patients with MUI; further studies should use uniform clinical efficacy criteria and gradually establish a homogeneous set of outcome indicators directly related to patient benefits. (3) Control groups of EA and sham EA should be set to confirm the placebo effect. (4) Given the mixed nature and complexity of MUI, further research should focus on classifying mixed incontinence in women into more discrete subtypes, which will ultimately provide the basis for more personalized clinical treatment recommendations. (5) Standardized acupuncture techniques should be used in clinical treatment, such as using uniform base acupoints, acupuncture depth, frequency and waveform of electroacupuncture, and treatment duration. This does not contradict individualized treatment, but prevents disunity in acupuncture treatment, thus facilitating the comparison of evidence, reducing clinical heterogeneity, and improving the level of evidence.

## Conclusions

Based on evidence from this analysis, compared with antimuscarinic drugs combined with PFMT, EA therapy has the potential of reducing urine leakage, as assessed with the 1-h pad test and ICIQ-SF scores in patients with MUI, although the difference in the 72-h IEF results was not statistically significant. Moreover, EA therapy is relatively safe and has minimal side effects. Evidence suggests that EA therapy can be considered in the treatment of MUI as a complementary and auxiliary therapy. Nevertheless, because of the limitations of this study, our conclusions should be interpreted with caution, and further studies are needed to confirm the comprehensive clinical efficacy and placebo effect of EA.

## Data Availability

The original data, appendix, and references used to support the findings of this study are available from the first and the corresponding authors upon request.
